# Associations between intimate partner violence and reproductive and maternal health outcomes in Bihar, India: a cross-sectional study

**DOI:** 10.1186/s12978-018-0551-2

**Published:** 2018-06-19

**Authors:** Diva Dhar, Lotus McDougal, Katherine Hay, Yamini Atmavilas, Jay Silverman, Daniel Triplett, Anita Raj

**Affiliations:** 1 Bill & Melinda Gates Foundation, New Delhi, India; 2 Center on Gender Equity and Health, Department of Medicine, University of California, San Diego School of Medicine, 9500 Gilman Drive, MC 0507, La Jolla, San Diego, CA 92093-0507 USA

**Keywords:** Intimate partner violence, Physical abuse, Sexual abuse, Reproductive health, Stillbirth, Miscarriage, Induced abortion, Delivery complications, Pregnancy complications

## Abstract

**Background:**

Bihar, India has higher rates of intimate partner violence (IPV) and maternal and infant mortality relative to India as a whole. This study assesses whether IPV is associated with poor reproductive and maternal health outcomes, as well as whether poverty exacerbates any observed associations, among women who gave birth in the preceding 23 months in Bihar, India.

**Methods:**

A cross-sectional analysis of data from a representative household sample of mothers of children 0–23 months old in Bihar, India (*N* = 13,803) was conducted. Associations between lifetime IPV (physical and/or sexual violence) and poor reproductive health outcomes ever (miscarriage, stillbirth, and abortion) as well as maternal complications for the index pregnancy (early and/or prolonged labor complications, other complications during pregnancy or delivery) were assessed using multivariable logistic regression, adjusting for demographics and fertility history of the mother. Models were then stratified by wealth index to determine whether observed associations were stronger for poorer versus wealthier women.

**Results:**

IPV was reported by 45% of women in the sample. A history of miscarriage, stillbirth, and abortion was reported by 8.7, 4.6, and 1.3% of the sample, respectively. More than one in 10 women (10.7%) reported labor complications during the last pregnancy, and 16.3% reported other complications during pregnancy or delivery. Adjusted regressions revealed significant associations between IPV and miscarriage (AOR = 1.35, 95% CI = 1.11–1.65) and stillbirth (AOR = 1.36, 95% CI = 1.02–1.82) ever, as well as with labor complications (AOR = 1.27, 95% CI = 1.04–1.54) and other pregnancy/delivery complications (AOR = 1.68, 95% CI = 1.42–1.99). Women in the poorest quartile (Quartile 1) saw no associations between IPV and miscarriage (Quartile 1 AOR = 0.98, 95% CI = 0.67–1.45) or stillbirth (Quartile 1 AOR = 1.17, 95% CI = 0.69–1.98), whereas women in the higher wealth quartile (Quartile 3) did see associations between IPV and miscarriage (Quartile 3 AOR = 1.55, 95% CI = 1.07, 2.25) and stillbirth (Quartile 3 AOR = 1.79, 95% CI = 1.04, 3.08).

**Discussion:**

IPV is highly prevalent in Bihar and is associated with increased risk for miscarriage, stillbirth, and maternal health complications. Associations between IPV and miscarriage and stillbirth do not hold true for the poorest women, possibly because other risks attached to poverty and deprivation may be greater contributors.

## Plain English summary

Intimate partner violence (IPV) can increase maternal health concerns. This study assessed the prevalence of intimate physical and/or sexual IPV among mothers who gave birth in the past 2 years in Bihar, India. In addition, it examined the association of IPV with increased risk of poor birth outcomes (e.g., miscarriage and stillbirth) ever or of pregnancy and delivery complications in the most recent birth. Almost half of these mothers (43%) were physically and/or sexually abused by their husbands in the past year, that is, in the perinatal period for this sample. Further, a history of such violence increased health risks, including miscarriage, stillbirth, and maternal complications.

The study also explored the intersectionality of IPV and poverty with pregnancy and birth outcomes. The association of IPV with miscarriage and stillbirth was less likely to hold true for the richest and poorest women, possibly due to the extremes of high resources (for the richest) and deprivation (for the poorest) being more important contributors to fetal health and survival. However, IPV was associated with labor and delivery complications regardless of women’s income level. Notably, mobile phone ownership was protective for stillbirth.

Overall, these findings highlight the importance of maternal health interventions to address potential health concerns related to the physical and sexual intimate partner violence women face, as a means of improving maternal and infant health outcomes. However, they also suggest the potential utility of asset ownership in conjunction with health approaches, to produce better health outcomes from an empowerment perspective.

## Background

Intimate partner violence (IPV) against women is a pervasive human rights and public health concern [1, 2]. Globally, one third of women (35%) have faced physical and/or sexual IPV at some point in their lives [2, 3]. Robust multi-country analyses and meta-analyses document associations between IPV and poor reproductive health outcomes such as miscarriage and abortion at a global scale [1, 3–5]. This is particularly important for South Asia, as more than 1 in 4 of the world’s pregnancies [6], and thus a likely disproportionate burden of poor pregnancy outcomes, occurs in the region, and South Asia maintains some of the highest rates of IPV seen globally [2, 3].

Studies from India, South Asia’s largest and most populous nation, also indicate high prevalence of poor reproductive health outcomes, with little improvement in the past decade [11]. State and multi-state level data from women in India indicate a significant association between IPV and abortion [7, 8]. Studies from within the states of Uttar Pradesh and Maharashtra indicate that IPV is also associated with maternal health complications [9, 10]. Nationally-representative data disaggregating miscarriage and abortion are not yet available in India to assess potential associations between IPV and these outcomes, and research on IPV and miscarriage and stillbirth outcomes in India could not be identified in the literature. Much of this work is older, and state level analyses have not included a focus on Bihar, a populous Indian state that has both higher prevalence of IPV (43% for Bihar vs. 29% for India) [11] and maternal mortality ratio (208 for Bihar vs 167 for India, per 100,000 births) [12], relative to India as a whole.

Higher vulnerability to IPV and related health outcomes in Bihar may be a consequence of poorer development and infrastructure in the state [11]. We also see poorer antenatal care utilization and use of important nutritional supplements during pregnancy, as well as higher unmet need for family planning, for Bihar relative to India [11], and these factors are both linked to IPV and can compromise reproductive and maternal health outcomes [1, 3]. These are also tied to poverty in India, which is additionally linked to increased risk for IPV [13]. Hence, poverty may help explain or even exacerbate these associations between IPV and poor reproductive health outcomes. Exploration of this issue analytically has received inadequate attention from the research literature.

The present study builds upon existing evidence by assessing the relationship between lifetime experience of IPV and reproductive health outcomes (miscarriage, stillbirth, and abortion) and maternal health complications among women in Bihar, India. Previous studies have been limited by reliance on older data and have not delineated the association of IPV with different types of pregnancy outcomes such as stillbirth and miscarriage. For maternal complications, we have disaggregated prolonged labor from other complications in pregnancy or delivery given findings from a prospective multicountry study inclusive of India that documents that these complications are on the causal pathway for increased risk for maternal mortality, stillbirth, and neonatal mortality [14]. Secondarily, we also contribute to the literature by exploring the intersectionality of IPV and poverty for women in Bihar, in terms of their potentially greater combined impact on these same reproductive and maternal health outcomes. Findings from this work can guide interventions that seek to simultaneously advance maternal and child health and gender equality.

## Methods

### Study design

We conducted a cross-sectional analysis of follow-up data from the Ananya evaluation, collected via household surveys from March to June 2014 with women who had given birth in the past 23 months in Bihar, India. Ananya was a public health program that supported a combination of supply-side and demand generation efforts to increase maternal and child health care utilization via the public health system in Bihar; the program and corresponding evaluation were initiated in 2012 to address persistent challenges in reproductive, maternal, newborn and child health in the state [15]. Details on the program and evaluation findings are published elsewhere [15, 16]. Ananya was not designed to identify or intervene on issues of IPV, but IPV was included in the 2014 follow-up survey.

#### Sampling and procedure

A sample of mothers representative at the state-level who delivered in the past 23 months was included in the current study; this included the eight districts in Bihar in which the Ananya program was implemented as well as the remaining 30 districts assigned to the standard of care control condition for the evaluation. Details on the study’s multi-stage sampling and recruitment procedures are available elsewhere [15, 16]. The 2014 follow-up survey had a response rate of 87%, and yielded 11,408 interviews with mothers who had children aged 0–11 months, and 2455 interviews with mothers who had children aged 12–23 months.

Given the sensitive nature of the information, all data were collected by trained female study staff, after obtaining written informed consent. The staff were trained in sensitive assessment according to WHO guidelines [17], ensuring complete privacy during questions and a debriefing in cases where trauma or retraumatization were indicated. Referrals were provided to local domestic violence service. If indications of immediate lethal risk was indicated, the survey was to be stopped and immediate connection to protections was to be provided. However, no such incidents occurred. Ethical approval for the original evaluation study was provided by the Screening Committee of Government of India’s Ministry of Health and Family Welfare. Ethical approval for this analysis was provided by the University of California, San Diego (UCSD). The current analysis was restricted to women with singleton births and without missing values for any variables of interest (*n* = 13,803, a loss of only *n* = 63 participants in the dataset).

#### Measures

The independent variable for this analysis was self-reported lifetime experiences of physical and/or sexual intimate partner violence in their current marriage. Physical violence was comprised of items on whether the participants had experienced any of the following from her husband: being slapped, having an arm twisted or hair pulled, being pushed with a fist, being shaken or having something thrown at them, being kicked, dragged or beaten up, or choking or burning. Sexual violence was measured using a single item asking “Did your husband ever physically force you to have sexual intercourse with him even when you did not want to?” Responses were yes/no for the timeframes of ever and past 12 months. Both any IPV ever and IPV recent measures were created from these items, but only the IPV ever measure was included as the independent variable given that multiple outcomes of focus were also in this timeframe. These IPV items were validated in the multi-country study on Violence Against Women by the World Health Organization and are routinely included in the Demographic Health Surveys (DHS), including in India’s DHS, known as the National Family Health Survey (NFHS) [1, 13].

Dependent variables included the following reproductive health outcomes: miscarriages, stillbirths, abortions; these were assessed via single survey items on whether these ever occurred. Maternal health complications were specific to the index childbirth that allowed them to meet eligibility criteria, which was the most recent childbirth occurring in the past 23 months, and these were divided into labor complications and symptoms of other pregnancy or delivery complications. Items on labor complications assessed prolonged labor over 12 h and pre-term labor, defined as labor pain in the seventh or eighth month of pregnancy. Items on other pregnancy or delivery complications included excessive bleeding, convulsions, swelling of the hands, body, or face, fever, vaginal discharge, or “any other such symptom”.

Covariates considered in the analyses related to sociodemographics, parity, and study design. Sociodemographics assessed included maternal age (15 to 19, 20 to 24, 25 to 29, 30 or older), wealth (in quartiles), education (none, primary, secondary or higher), religion and caste identity (Scheduled Tribe/Scheduled Caste [SC/ST], Other Backward Caste [OBC], Muslim, or General Caste], and paternal education (none, primary, secondary or higher). The wealth index was constructed via principle component analysis of household characteristics and assets following the technique used in the NFHS [13]. Parity at survey was categorized as 1, 2, or 3 or more. The study design covariate simply accounted for Ananya treatment group (i.e., whether or not the respondent lived in an Ananya program district).

Given the study focus on IPV, we additionally considered gender equity variables as covariates. These included maternal age at marriage (under 18 vs. 18 or older), workforce participation in the past 12 months, personal bank account ownership, and ownership of a mobile phone, as they are factors related to autonomy that are significantly different for women and men in India [11]. For maternal complication outcomes, we also included covariates specific to the index child: sex of child, age of child (0 to 5 months, 6 to 11 months, 12 to 23 months), having four or more antenatal care visits, and having a skilled birth attendant at delivery.

### Data analysis

Descriptive frequencies were calculated for all outcomes and covariates, cross-tabulated by lifetime IPV with Rao-Scott chi-square tests of independence. Univariate and multivariable logistic regression models were used to assess the associations between IPV on each health outcome. Although Ananya district, age of mother, and parity were forced into all adjusted models, backwards stepwise model building was conducted for each outcome using an inclusion cut point of *p* < 0.2. Institutional versus home delivery was also considered for inclusion, but 97% of those with a skilled birth attendant delivered in an institution and the variance inflation factors exceeded 6.5 in a collinearity check, so these variables were deemed overlapping. This approach was used to allow for a more parsimonious model, given the small cell sizes for some outcomes such as abortion. An additional model was conducted to test for an interaction between IPV and wealth. Final parsimonious models for all outcomes were then stratified by wealth index to provide insight into the intersectionality of IPV and poverty. All analyses adjusted for survey design and individual sampling weights and were conducted using Stata 14.2 SE; and statistical significance was evaluated at *p* < .05 unless otherwise indicated.

## Results

### Description of the sample

Participants were aged 15–49 years (Mean = 25.3, Std Dev = 4.3). The majority (52.1%) had no formal education, and were from a socially marginalized group (as indicated by 26.5% SC/ST, 46.3% OBC, and 17.3% Muslim). [See Table 1.] For the index pregnancy, 30.1% were first children, and 53.2% were male. Receipt of four or more antenatal care visits was reported by only 20.0% of the sample, but 72.4% had a skilled birth attendant at delivery.

**Table 1:** Descriptive Statistics by Lifetime Experience of IPV (*N* = 13,803)

	Total (*N* = 13,803)	Any IPV in Lifetime	No IPV Ever	*p*-value
	–	% [95% CI]	% [95% CI]	
	–	*N*	*N*	
Total	% [95% CI]	45.1 [43.2, 47.0]	54.9 [53.0, 56.8]	
	N	6256	7547	
Outcomes				
Miscarriage				<.001
No	91.3 [90.4, 92.1]	89.7 [88.2, 91.1]	92.6 [91.5, 93.5]	
	12,634	5657	6977	
Yes	8.7 [7.9, 9.6]	10.3 [8.9, 11.8]	7.4 [6.5, 8.5]	
	1169	599	570	
Stillbirth				<.001
No	95.4 [94.7, 96.0]	94.2 [93.1, 95.2]	96.4 [95.6, 97.0]	
	13,194	5915	7279	
Yes	4.6 [4.0, 5.3]	5.8 [4.8, 6.9]	3.6 [3.0, 4.4]	
	609	341	268	
Abortion				.506
No	98.7 [98.3, 99.1]	98.9 [98.4, 99.2]	98.6 [97.9, 99.1]	
	13,658	6174	7484	
Yes	1.3 [0.9, 1.7]	1.1 [0.8, 1.6]	1.4 [0.9, 2.1]	
	145	82	63	
Labor Complications				.200
No	89.3 [88.2, 90.3]	88.6 [87.0, 90.0]	89.8 [88.4, 91.1]	
	12,364	5551	6813	
Yes	10.7 [9.7, 11.8]	11.4 [10.0, 13.0]	10.2 [8.9, 11.6]	
	1439	705	734	
Other Pregnancy or Delivery Complications				<.001
No	83.7 [82.3, 85.0]	80.5 [78.5, 82.4]	86.3 [84.7, 87.7]	
	11,660	5101	6559	
Yes	16.3 [15.0, 17.7]	19.5 [17.6, 21.5]	13.7 [12.3, 15.3]	
	2143	1155	988	
Sociodemographics, parity, study design				
Age				
15–19	27.7 [26.4, 29.1]	24.3 [22.5, 26.1]	30.6 [28.7, 32.5]	<.001
	4004	1621	2383	
20–24	33.0 [31.7, 34.4]	32.8 [30.9, 34.8]	33.2 [31.3, 35.1]	
	4581	2045	2536	
25–29	20.1 [18.8, 21.6]	21.0 [19.4, 22.8]	19.4 [17.6, 21.4]	
	2764	1319	1445	
35+	19.1 [17.8, 20.5]	21.9 [20.2, 23.6]	16.9 [15.1, 18.8]	
	2454	1271	1183	
Wealth quartile				<.001
1 (poorest)	28.5 [26.4, 30.7]	32.7 [30.0, 35.5]	25.1 [22.8, 27.5]	
	3692	1934	1758	
2	20.3 [19.1, 21.7]	21.1 [19.2, 23.1]	19.7 [18.2, 21.3]	
	2761	1316	1445	
3	24.2 [22.8, 25.6]	24.5 [22.7, 26.5]	23.9 [22.0, 25.9]	
	3271	1573	1698	
4 (wealthiest)	27.0 [25.1, 29.0]	21.7 [19.6, 23.9]	31.3 [28.9, 33.9]	
	4079	1433	2646	
Education				<.001
None	52.1 [50.1, 54.1]	61.0 [58.6, 63.3]	44.8 [42.4, 47.2]	
	6929	3667	3262	
Primary	26.0 [24.5, 27.5]	23.7 [21.9, 25.7]	27.8 [25.9, 29.9]	
	3561	1544	2017	
Secondary	21.9 [20.5, 23.4]	15.3 [13.7, 17.0]	27.4 [25.4, 29.4]	
	3313	1045	2268	
Caste/religion				<.001
SC/ST	26.5 [24.2, 28.9]	31.2 [28.3, 34.4]	22.6 [20.0, 25.3]	
	3538	1980	1558	
OBC	46.3 [43.6, 49.0]	44.3 [41.2, 47.4]	47.9 [44.7, 51.2]	
	6481	2810	3671	
General	10.0 [8.7, 11.5]	8.1 [6.5, 10.1]	11.5 [10.0, 13.2]	
	1416	458	958	
Muslim	17.3 [14.6, 20.2]	16.4 [13.7, 19.5]	18.0 [14.9, 21.5]	
	2368	1008	1360	
Ananya district				.070
No	73.4 [70.3, 76.2]	75.2 [71.8, 78.3]	71.9 [68.1, 75.4]	
	10,185	4685	5500	
Yes	26.6 [23.8, 29.7]	24.8 [21.7, 28.2]	28.1[24.6, 31.9]	
	3618	1571	2047	
Spouse’s Education				<.001
None	31.1 [29.2, 33.0]	36.3 [33.8, 38.8]	26.8 [24.6, 29.1]	
	4161	2233	1928	
Primary	34.4 [32.9, 35.9]	35.7 [33.8, 37.7]	33.2 [31.3, 35.3]	
	4564	2155	3409	
Secondary	34.6 [32.6, 36.6]	28.0 [25.7, 30.4]	40.0 [37.4, 42.6]	
	5078	1868	3210	
Parity				<.001
1	30.1 [28.8, 31.5]	25.3 [23.6, 27.2]	34.1 [32.2, 36.0]	
	4306	1612	2694	
2	27.3 [26.1, 28.6]	27.4 [25.6, 29.4]	27.2 [25.7, 28.8]	
	3897	1751	2146	
3+	42.6 [41.0, 44.2]	47.2 [45.0, 49.4]	38.7 [36.6, 40.9]	
	5600	2893	2707	
Gender equity				
Age at marriage				<.001
< 18	46.8 [44.8, 48.8]	53.6 [50.8, 56.3]	41.2 [38.9, 43.5]	
	6099	3250	2849	
18+	53.2 [51.2, 55.2]	46.4 [43.7, 49.2]	58.8 [56.5, 61.1]	
	7704	3006	4698	
Workforce participation, past 12 months				<.001
No	94.0 [93.0, 94.9]	91.7 [90.1, 93.1]	95.9 [94.9, 96.8]	
	13,059	5798	7261	
Yes	6.0 [5.1, 7.0]	8.3 [6.9, 10.0]	4.1 [3.2, 5.1]	
	744	458	286	
Personal bank account				.034
No	71.1 [69.3, 72.8]	72.8 [70.4, 75.0]	69.7 [67.5, 71.8]	
	9619	4508	5111	
Yes	28.9 [27.2, 30.7]	27.2 [25.0, 29.6]	30.3 [28.2, 32.5]	
	4152	1736	2416	
Personal mobile phone				<.001
No	34.4 [32.6, 36.2]	40.0 [37.3, 42.8]	29.7 [27.6, 32.0]	
	4720	2436	2284	
Yes	65.6 [63.8, 67.4]	60.0 [57.2, 62.7]	70.3 [68.0, 72.4]	
	9083	3820	5263	
Focus child characteristics				
Gender of focal child				.832
Female	46.8 [45.3, 48.4]	47.0 [44.8, 49.3]	46.7 [44.5, 48.8]	
	6385	2889	3496	
Male	53.2 [51.6, 54.7]	53.0 [50.7, 55.2]	53.3 [51.2, 55.5]	
	7418	3367	4051	
Age of child (months)				.200
0–5 months	36.8 [35.8, 37.9]	36.7 [35.2, 38.3]	36.9 [35.3, 38.6]	
	6582	2960	3622	
6–11 months	26.3 [25.4, 27.3]	27.5 [26.0, 29.0]	25.4 [24.1, 26.8]	
	4781	2214	2567	
12–23 months	36.8 [35.6, 38.0]	35.8 [33.7, 38.0]	37.7 [35.8, 39.6]	
	2440	1082	1358	
4 or more antenatal care visits				<.001
No	80.0 [78.7, 81.3]	83.7 [82.1, 85.2]	77.0 [75.1, 78.8]	
	10,920	5160	5760	
Yes	20.0 [18.7, 21.3]	16.3 [14.8, 17.9]	23.0 [21.2, 24.9]	
	2883	1096	1787	
Skilled birth attendance				<.001
No	27.6 [25.9, 29.3]	30.6 [28.2, 33.1]	25.2 [23.2, 27.2]	
	3653	1860	1793	
Yes	72.4 [70.7, 74.1]	69.4 [66.9, 71.8]	74.8 [72.8, 76.8]	
	10,150	4396	5754	

Almost half of participants (45.1%) reported IPV ever; 28.6% reported only physical IPV, while 2.3% of them had faced only sexual IPV and 14.3% had faced both physical and sexual IPV. [See Table 1.] A slightly smaller percentage (43.0%) had experienced IPV in the past 12 months, with 27.6% reporting only recent physical IPV, 2.7% reporting only recent sexual IPV only, and 12.7% reporting both. A history of miscarriage, stillbirth, and abortion was reported by 8.7, 4.6, and 1.3% of the sample, respectively. More than one in 10 women (10.7%) reported labor complications at last pregnancy, and 16.3% reported other complications during pregnancy or delivery.

### Correlates of IPV

Chi-square tests document significant associations (*P* < .001) between IPV and each of the following outcomes: miscarriage, stillbirth, and other pregnancy or delivery complications [Table 1]. However, upon breaking down other pregnancy and delivery complications into its component types, it appeared that excessive bleeding, convulsions, and swelling may have been the drivers of this association. Though 4.6% of the sample reported bleeding, another 4.6% convulsions, 6.5% swelling, 6.2% fever, 2.9% vaginal discharge, and 1.4% another symptom, the women who had endured partner violence reported significantly higher proportions of these first three relative to those who had not (bleeding: 5.7% vs. 3.7%, *P* < .001; convulsions: 5.6% vs. 3.9% *P* = .02; swelling: 6.1% vs. 5.1%, *P* < .001). Moreover, 8.6% of the sample reported prolonged labor and 3.5% reported pre-term labor, but these did not differ by experience of IPV.

Chi-square analyses also indicated that women reporting IPV were significantly more likely to indicate greater sociodemographic vulnerability including low to no education, a husband with low to no education, and early marriage. (See Table 1.) IPV was also more likely among older women and those with higher parity, as well as those not reporting 4+ ANC visits or a skilled birth attendant (SBA) for the index pregnancy. In terms of economic indicators, IPV was associated with lesser wealth, lack of female work force participation in the last 12 months, and not having a bank account or phone, which were all significantly correlated with each other.

### Regression analyses assessing associations between IPV with reproductive and maternal health outcomes

Adjusted logistic regression analysis revealed that women who had ever faced IPV in their relationships had higher odds of ever having had a miscarriage (AOR = 1.35, 95% CI = 1.11–1.65) or stillbirth (AOR = 1.36, 95% CI = 1.02–1.82). (See Table 2.) Women’s risk for ever having these outcomes increased with age, as expected. Women who married at age 18 or older (relative to married at < 18) had lower odds of miscarriage and stillbirth. Women with more educated husbands had higher odds of stillbirth, whereas those who owned a mobile phone had lower odds of stillbirth ever. No significant association between IPV and abortion was observed, but the wealthiest women (relative to the poorest) and employed women (relative to non-income generating women) had higher odds of ever having had abortion, whereas women who married at age 18 or older (relative to those married at < 18) and those of marginalized caste/religion (relative to general caste women) had lower odds of having received an abortion.

**Table 2:** Logistic Regression to Assess Associations between Lifetime Experience of IPV and Birth Outcomes

	Unadjusted			Adjusted		
Variables	Miscarriage	Stillbirth	Abortion	Miscarriage (*n* = 13,803)	Stillbirth (*n* = 13,771)	Abortion (*n* = 13,803)
Physical or Sexual IPV						
Never	REF	REF	REF	REF	REF	REF
Ever	1.42 (1.16–1.74)	1.62 (1.22–2.15)	0.83 (0.47–1.45)	1.35 (1.11–1.65)	1.36 (1.02–1.82)	0.76 (0.40–1.44)
Age						
15–19	REF	REF	REF	REF	REF	REF
20–24	1.70 (1.30–2.23)	1.48 (0.95–2.30)	1.75 (0.73–4.20)	1.61 (1.22–2.11)	1.42 (0.88–2.30)	1.68 (0.67–4.23)
25–29	1.72 (1.25–2.37)	2.33 (1.53–3.54)	2.62 (1.03–6.62)	1.54 (1.08–2.18)	2.27 (1.36–3.79)	2.40 (0.90–6.43)
30+	2.51 (1.87–3.38)	2.68 (1.74–4.13)	1.48 (0.61–3.56)	2.17 (1.54–3.05)	2.65 (1.57–4.46)	1.40 (0.50–3.91)
Wealth Quartile						
1 (Poorest)	REF	REF	REF	–	–	REF
2	1.02 (0.75–1.38)	0.74 (0.49–1.13)	1.28 (0.46–3.54)			1.28 (0.46–3.59)
3	1.03 (0.77–1.36)	0.82 (0.56–1.19)	3.36 (1.36–8.30)			3.39 (1.26–9.16)
4 (Wealthiest)	0.93 (0.70–1.24)	0.59 (0.40–0.85)	2.50 (1.05–5.99)			2.80 (1.12–6.98)
Education						
None	REF	REF	REF	–	–	–
Primary	0.98 (0.74–1.30)	0.61 (0.44–0.85)	1.01 (0.51–2.02)			
Secondary	0.73 (0.55–0.97)	0.55 (0.38–0.81)	1.54 (0.76–3.11)			
Caste/Religion						
General	REF	REF	REF	REF	REF	REF
SC/ST	1.02 (0.65–1.59)	1.25 (0.82–1.90)	0.29 (0.09–0.91)	0.87 (0.56–1.37)	1.01 (0.67–1.54)	0.28 (0.10–0.81)
OBC	1.06 (0.70–1.61)	0.78 (0.53–1.14)	0.44 (0.21–0.92)	0.98 (0.65–1.47)	0.68 (0.46–1.00)	0.41 (0.21–0.80)
Muslim	1.47 (0.90–2.40)	0.99 (0.61–1.60)	0.33 (0.13–0.81)	1.26 (0.78–2.03)	0.79 (0.48–1.28)	0.29 (0.12–0.69)
Spouse’s education						
None	REF	REF	REF	REF	REF	–
Primary	1.13 (0.89–1.44)	1.19 (0.89–1.60)	0.99 (0.41–2.43)	1.27 (1.00–1.61)	1.46 (1.08–1.99)	
Secondary	0.92 (0.71–1.18)	0.84 (0.60–1.17)	1.61 (0.69–3.75)	1.17 (0.89–1.52)	1.33 (0.92–1.91)	
Parity						
1	REF	REF	REF	REF	REF	REF
2	1.55 (1.18–2.04)	2.00 (1.33–2.99)	1.51 (0.70–3.24)	1.22 (0.94–1.59)	1.48 (0.94–2.32)	1.16 (0.52–2.58)
3+	1.90 (1.47–2.46)	2.17 (1.60–2.93)	1.67 (0.81–3.46)	1.17 (0.86–1.58)	1.04 (0.70–1.57)	1.26 (0.58–2.71)
Ananya district						
No	REF	REF	REF	REF	REF	REF
Yes	1.37 (1.07–1.75)	1.04 (0.75–1.44)	1.52 (0.80–2.90)	1.38 (1.09–1.75)	0.99 (0.71–1.37)	1.47 (0.83–2.60)
GENDER EQUITY						
Age at marriage						
< 18	REF	REF	REF	REF	REF	REF
18+	0.68 (0.55–0.83)	0.46 (0.35–0.61)	0.51 (0.29–0.91)	0.74 (0.61–0.91)	0.51 (0.38–0.68)	0.43 (0.23–0.79)
Workforce participation, past 12 months						
No	REF	REF	REF	–	–	REF
Yes	0.96 (0.62–1.47)	1.02 (0.64–1.63)	2.24 (1.03–4.89)			2.57 (1.17–5.65)
Personal bank account						
No	REF	REF	REF	–	REF	–
Yes	0.93 (0.75–1.16)	0.74 (0.53–1.03)	1.64 (0.84–3.21)		0.74 (0.53–1.04)	
Personal mobile phone						
No	REF	REF	REF	–	REF	–
Yes	0.98 (0.80–1.20)	0.66 (0.49–0.89)	0.98 (0.55–1.72)		0.73 (0.54–0.99)	

Multivariable analyses also documented significant associations between IPV and both labor complications at last birth (AOR = 1.27, 95% CI = 1.04–1.54), and other pregnancy or delivery complications at last birth (AOR = 1.68, 95% CI = 1.42–1.99). (See Table 3.) Notably, the bivariate association between IPV and labor complications was not significant, but became significant in adjusted analyses. Women had lower odds of labor complications if they were older and were higher parity, but women with more education, an educated husband, employment, a male child, and receipt of ANC and an SBA had higher odds of labor complications. In terms of complications during delivery, women who were older and those with a mobile phone had lower odds of this outcome, though again husband education, women’s employment, receipt of ANC and SBA, as well as a male index child were associated with higher odds of complications during delivery.

**Table 3:** Logistic Regression to Assess Associations between Lifetime Experience of IPV and Labor/Pregnancy Complications

	Unadjusted		Adjusted	
Variables	Labor complications	Pregnancy or delivery complications	Labor complications	Pregnancy or delivery complications
Physical or Sexual IPV				
Never	REF	REF	REF	REF
Ever	1.14 (0.93–1.38)	1.52 (1.29–1.78)	1.27 (1.04–1.54)	1.68 (1.42–1.99)
Age				
15–19	REF	REF	REF	REF
20–24	0.83 (0.66–1.05)	0.70 (0.59–0.83)	0.91 (0.72–1.17)	0.70 (0.58–0.85)
25–29	0.59 (0.44–0.79)	0.68 (0.54–0.86)	0.71 (0.51–0.99)	0.68 (0.52–0.89)
30+	0.70 (0.52–0.93)	0.74 (0.57–0.96)	0.95 (0.67–1.34)	0.78 (0.56–1.10)
Wealth Quartile				
1 (Poorest)	REF	REF	REF	–
2	0.76 (0.56–1.03)	1.02 (0.79–1.33)	0.74 (0.55–1.00)	
3	0.87 (0.65–1.17)	0.97 (0.75–1.26)	0.78 (0.58–1.04)	
4 (Wealthiest)	1.23 (0.91–1.67)	1.24 (0.95–1.61)	0.95 (0.69–1.31)	
Education				
None	REF	REF	REF	REF
Primary	1.20 (0.94–1.54)	1.21 (1.00–1.47)	1.26 (1.05–1.50)	1.08 (0.92–1.28)
Secondary	1.43 (1.12–1.83)	1.31 (1.06–1.63)	1.38 (1.14–1.66)	1.24 (0.99–1.55)
Caste/Religion				
General	REF	REF	REF	–
SC/ST	0.88 (0.61–1.29)	0.86 (0.61–1.20)	0.84 (0.63–1.14)	
OBC	0.99 (0.70–1.41)	0.96 (0.71–1.30)	0.93 (0.71–1.20)	
Muslim	0.98 (0.66–1.46)	0.98 (0.70–1.36)	0.94 (0.70–1.25)	
Spouse’s education				
None	REF	REF	REF	REF
Primary	1.34 (1.03–1.74)	1.18 (0.96–1.45)	1.32 (1.03–1.69)	1.17 (0.95–1.43)
Secondary	1.46 (1.13–1.90)	1.33 (1.07–1.65)	1.24 (0.95–1.62)	1.28 (1.02–1.62)
Parity				
1	REF	REF	REF	REF
2	0.83 (0.66–1.05)	0.91 (0.74–1.11)	0.95 (0.76–1.19)	1.06 (0.84–1.33)
3+	0.60 (0.48–0.74)	0.78 (0.63–0.95)	0.75 (0.59–0.95)	0.97 (0.74–1.27)
Ananya district				
No	REF	REF	REF	REF
Yes	1.27 (0.99–1.63)	1.49 (1.21–1.84)	1.25 (0.97–1.60)	1.46 (1.19–1.80)
Gender equity				
Age at marriage				
< 18	REF	REF	REF	–
18+	1.29 (1.08–1.55)	0.96 (0.83–1.12)	1.18 (0.97–1.42)	
Workforce participation, past 12 months				
No	REF	REF	REF	REF
Yes	1.76 (1.09–2.82)	1.54 (1.10–2.14)	2.09 (1.32–3.31)	1.60 (1.14–2.24)
Personal bank account				
No	REF	REF	REF	–
Yes	0.96 (0.78–1.17)	1.03 (0.86–1.23)	0.85 (0.68–1.06)	
Personal mobile phone				
No	REF	REF	–	REF
Yes	1.02 (0.80–1.31)	0.89 (0.75–1.04)		0.86 (0.73–1.02)
Gender of focal child				
Female	REF	REF	REF	REF
Male	1.25 (1.04–1.51)	1.15 (0.96–1.38)	1.24 (1.02–1.49)	1.15 (0.96–1.38)
Age of focal child				
0–5 months	REF	REF	REF	–
6–11 months	0.86 (0.73–1.01)	1.03 (0.89–1.19)	0.85 (0.72–1.00)	
12–23 months	0.97 (0.78–1.21)	1.12 (0.91–1.39)	0.97 (0.77–1.21)	
4 or more antenatal care visits				
`No	REF	REF	REF	REF
Yes	1.68 (1.35–2.08)	1.81 (1.52–2.17)	1.50 (1.20–1.89)	1.73 (1.43–2.10)
Skilled birth attendance				
No	REF	REF	REF	REF
Yes	2.00 (1.59–2.51)	1.61 (1.33–1.97)	1.77 (1.41–2.24)	1.46 (1.19–1.79)

### Interaction effects of wealth index and IPV on reproductive and maternal health outcomes

To assess whether wealth moderated the effect of IPV on reproductive and maternal health outcomes, a wealth x IPV interaction term was included in the final parsimonious models described above, and wealth was restored to the model if it was otherwise dropped, to determine if the interaction affected our outcomes of interest. No interactions were observed at *p* < .15. Wealth stratified adjusted regression models were also conducted to assess whether observed associations between IPV and reproductive and maternal health outcomes differed by wealth quartile. Only miscarriage and stillbirth indicated differences in associations across wealth quartiles. (See Table 4.) IPV was not associated with miscarriage for poorest (Quartile 1) women (AOR = 0.98, 95% CI = 0.67–1.45), but significant IPV and miscarriage associations were seen with women in wealth quartiles 2 (AOR = 1.86, 95% CI = 1.19–2.92) and 3 (AOR = 1.55, 95% CI = 1.07–2.25). For stillbirth, a significant IPV effect was only seen for women in wealth Quartile 3 (AOR = 1.79, 95% CI = 1.04–3.08). Stratified analyses did not indicate meaningful differences in IPV effects on other outcomes by wealth quartile.

**Table 4:** Wealth-Stratified Logistic Regression Analyses Assessing Associations between IPV and Birth and Maternal Complication Outcomes

Equity Subgroup	Miscarriage^1^	Stillbirth^2^	Abortion^3^	Labor Complications^4^	Other Pregnancy or Delivery Complications^5^
Wealth Quartile					
Quartile 1 (Poorest) (*n* = 3692)	0.98 (0.67–1.45)	1.17 (0.69–1.98)	0.79 (0.22–2.88)	1.29 (0.84–1.98)	1.49* (1.03–2.16)
Quartile 2 (*n* = 2761)	1.86 (1.19–2.92)	1.17 (0.65–2.09)	1.68 (0.31–9.00)	1.25 (0.80–1.93)	2.03 (1.39–2.96)
Quartile 3 (*n* = 3271)	1.55 (1.07–2.25)	1.79 (1.04–3.08)	0.51 (0.21–1.25)	1.28 (0.93–1.75)	1.95 (1.44–2.64)
Quartile 4 (Wealthiest) (*n* = 4079)	1.31 (0.91–1.88)	1.40 (0.81–2.40)	0.86 (0.24–3.02)	1.27 (0.91–1.77)	1.49 (1.13–1.95)

1. Miscarriage adjusted for age of mother, SC/ST or Muslim, spousal education, parity, Ananya/non-Ananya district, and age at marriage;

2. Stillbirth adjusted for age of mother, parity, Ananya/non-Ananya district, SC/ST or Muslim, spousal education, personal bank account, and personal mobile phone;

3. Abortion adjusted for age of mother, parity, Ananya/non-Ananya district, SC/ST or Muslim, wealth, age at marriage, and workforce participation;

4. Labor complications adjusted for age of mother, parity, Ananya/non-Ananya district, wealth, spousal education, age at marriage, workforce participation, personal bank account, gender of child, age of child, antenatal care, and skilled birth attendance;

5. Other pregnancy/delivery complications adjusted for age of mother, parity, Ananya/non-Ananya district, spousal education, workforce participation, personal mobile phone, gender of child, antenatal care, and skilled birth attendance

## Discussion

The findings from this study indicate that nearly half of women in Bihar, India (45%) reported physical and/or sexual IPV from their husbands, and most of these also reported IPV in the past year, which would be in the pregnancy or postpartum period for this representative sample of mothers. This prevalence is fairly comparable to that seen in recent state level data from Bihar, which reported that 43.2% of married women (15–49 years) had ever experienced spousal violence [11]. Further, those who experience such violence, are significantly more likely to report miscarriage, stillbirth, and abortion, as well as maternal health complications, compared to women who had not faced violence from their husbands. These findings highlight the importance of the health care sector in terms of screening for IPV and supporting women with IPV histories as they appear to experience a disproportionate burden of high risk pregnancy outcomes.

Consistent with findings from prior multi-country research not specific to India [1], this study demonstrates that exposure to IPV increases women’s odds of experiencing miscarriage, and extends this work by also documenting that there is a significant association between IPV and stillbirth. These findings, taken with prior research from India demonstrating associations between IPV and infant morbidity and mortality [18–21], suggest that IPV is an important factor in compromising fetal health. Similarly, current findings also document increased risk for maternal complications, results seen in prior research from India [9, 10]. Observed associations between IPV and reproductive and maternal health risks could be attributable to direct injury from IPV in pregnancy, though studies from India suggest that IPV declines with pregnancy [11, 22]. Rather it may be due to other forms of abuses that may continue in pregnancy (e.g., denial of rest or food) [10, 20, 23], or from the cumulative stress and mental health trauma caused by IPV, manifesting in physical health concerns [24, 25] and poor perinatal health outcomes such as preterm delivery, rupture of uterus, infection, placental abruption, fetal injury and death [26, 27]. Further research is needed to explore the mechanisms to explain these observed associations.

Contrary to prior research from India [7, 8] and elsewhere [1, 3–5], findings from this study do not document a significant association between IPV and abortion. The prevalence of abortion was very small in the current study (< 2%) and may have been too small to be able to detect the association; this self-reported abortion data may be an underestimate given stigma around the practice in India [28], particularly due to the laws related to sex-selected abortion. Given that the respondents were all women who had given birth in the preceding 23 months, the estimate is also not representative of the larger population of women in the state. Further research is needed to clarify these findings. Nonetheless, even with this small number of cases, analysis of covariates documents that economic indicators drive this outcome. Wealthiest and employed women are most likely to have had an abortion. This is likely because wealth can bring greater access to abortion services [29], particularly through increased women’s autonomy, mobility, and resource control [30, 31]. While women’s workforce participation in India is more likely among poorer women [13], such participation even among this poorer population suggests that pregnancies may be linked with perceptions of higher opportunity costs as a child can result in lost earnings. Similar findings on wealth, income generation and abortion have been documented in other studies from India [29, 32, 33].

Economic circumstances also affect observed associations between IPV and miscarriage and stillbirth, as seen in the wealth-stratified analyses of our study. Contrary to our hypothesis, the associations between IPV and miscarriage and stillbirth were not seen for the poorest women in our study. These findings suggest that potential effects of IPV on fetal health and survival may not be as influential as other factors related to deprivation, such as poor maternal nutritional status [34], lack of education, lack of skilled attendant at delivery, lack of consistent antenatal care, low birth weight, prematurity and previous stillbirths [35]. In contrast, the wealthiest women may be buffered somewhat from these potential IPV effects on fetal health, as indicated by insignificant or attenuated findings for this wealth stratum in this study. Notably, middle-income women were the population seen to be more affected by the associations of IPV with miscarriage and stillbirth. In the case of stillbirth, women’s asset ownership/access such as to a mobile phone was associated with lower risk, possibly because these women had facilitated access to health care, social support, or healthy behaviors in pregnancy via such means. Assets such as a bank account or a mobile phone were also associated with lower risk for IPV in this sample, findings seen in prior research as well [36, 37].

While study findings document the potential value of asset ownership/control as a means of helping women reduce their perinatal health risks, and show that women living with IPV are less likely to have these assets, women’s income generation is associated with both increased risk for IPV and increased risk for maternal health complications. These findings are consistent with other studies from India documenting greater vulnerability to IPV for working women and suggesting that income generation may not be a valid indicator of women’s economic empowerment in this context [38–42]. In contrast, research from India documents that women’s direct ownership or control over assets or household resources is associated with lower risk for IPV [38, 43], findings also seen in the current study; this suggests that these constructs are better indicators of women’s economic empowerment in India. Financial inclusion programs (e.g., microfinance/microloans [44, 45], bank accounts/digital accounts [38, 46]) and self-help groups supporting women’s engagement in these programs [44, 47, 48] may facilitate women’s asset ownership/control and help reduce IPV and related perinatal health outcomes beyond efforts that can be offered through the health sector.

Findings from this study should be considered in accordance with certain limitations. This study involved cross-sectional analysis of survey data from a midline evaluation of an intervention in a single state in India. Causality cannot be assumed, though use of a state-wide representative sample of mothers who recently have given birth (excluding stillbirths) does allow greater generalizability of findings to the state. We also adjusted for intervention group, to address the role of treatment as a confounder. Experience of IPV in this study is limited to self-reported physical and sexual violence, and does not include other manifestations like emotional and financial violence. These data also rely on participant self-reports of reproductive outcomes and IPV experiences, which could be subject to recall and social desirability bias. Social desirability bias could be more acute for sensitive items such as sexual IPV and abortion, and could lead to an under-reporting of these experiences. Future research should include longitudinal data to assess prospectively the impact of IPV on reproductive and maternal outcomes; data from medical records and across multiple states would also support assessment of validity and improve generalizability of findings.

## Conclusion

Findings from this representative sample of mothers who recently gave birth in Bihar India document that almost half (43%) have experienced IPV in the past year, suggesting that such violence in the perinatal period is pervasive. Further, findings indicate that a history of IPV in their marital relationship is associated with increased maternal health risks, including miscarriage, stillbirth, and maternal complications. While observed associations were less likely to hold true for the richest and poorest women in terms of miscarriage and stillbirth, possibly due to the extremes of high resources (for the richest) and deprivation (for the poorest) being a more important contributors to fetal health risk than was IPV, IPV was associated with labor and delivery complications regardless of women’s income level. Notably, indicators of asset ownership were also related to lower risk for IPV and perinatal health concerns. These findings overall highlight the importance of maternal health interventions that can support women’s marital safety and address potential health concerns related to the violence may face, as a means of improving maternal and infant health outcomes. However, they also suggest the promise of economic interventions in conjunction with health approaches to produce better health outcomes from an empowerment perspective.
